# A Huntingtin Peptide Inhibits PolyQ-Huntingtin Associated Defects

**DOI:** 10.1371/journal.pone.0068775

**Published:** 2013-07-04

**Authors:** Yoan Arribat, Nathalie Bonneaud, Yasmina Talmat-Amar, Sophie Layalle, Marie-Laure Parmentier, Florence Maschat

**Affiliations:** Institut de Génomique Fonctionnelle (IGF), CNRS-UMR5203, INSERM-U661, University of Montpellier, Montpellier, France; National Center of Neurology and Psychiatry, Japan

## Abstract

**Background:**

Huntington’s disease (HD) is caused by the abnormal expansion of the polyglutamine tract in the human Huntingtin protein (polyQ-hHtt). Although this mutation behaves dominantly, *huntingtin* loss of function also contributes to HD pathogenesis. Indeed, wild-type Huntingtin plays a protective role with respect to polyQ-hHtt induced defects.

**Methodology/Principal Findings:**

The question that we addressed here is what part of the wild-type Huntingtin is responsible for these protective properties. We first screened peptides from the Huntingtin protein in HeLa cells and identified a 23 aa peptide (P42) that inhibits polyQ-hHtt aggregation. P42 is part of the endogenous Huntingtin protein and lies within a region rich in proteolytic sites that plays a critical role in the pathogenesis process. Using a *Drosophila* model of HD, we tested the protective properties of this peptide on aggregation, as well as on different polyQ-hHtt induced neuronal phenotypes: eye degeneration (an indicator of cell death), impairment of vesicular axonal trafficking, and physiological behaviors such as larval locomotion and adult survival. Together, our results demonstrate high protective properties for P42 *in vivo*, in whole animals. These data also demonstrate a specific role of P42 on Huntington’s disease model, since it has no effect on other models of polyQ-induced diseases, such as spinocerebellar ataxias.

**Conclusions/Significance:**

Altogether our data show that P42, a 23 aa-long hHtt peptide, plays a protective role with respect to polyQ-hHtt aggregation as well as cellular and behavioral dysfunctions induced by polyQ-hHtt *in vivo*. Our study also confirms the correlation between polyQ-hHtt aggregation and neuronal defects. Finally, these results strongly suggest a therapeutic potential for P42, specific of Huntington’s disease.

## Introduction

In Humans, Huntington’s disease (HD) is an inherited neurodegenerative disorder belonging to the polyglutamine pathologies. This disease is caused by an abnormal expansion of the CAG triplet in the *huntingtin* gene (*htt*). When the number of CAG repeats exceeds 35, the resulting mutant protein (polyQ-Htt) forms intracellular aggregates and causes neuronal degeneration, particularly affecting the neurons of the striatum. Consistent with a toxic role of aggregates in the disease, aggregation increases with the progression of the disease in HD mouse model [1]. In particular, when polyQ-Htt expression is turned off, aggregates disappear, leading to an improvement in the behavioral and cognitive deficits in the HD mouse model [2]. In Humans, HD affects 5 out of 100,000 people, with symptoms usually appearing in patients by their late 40s. HD is a fatal neurological disease with no effective treatment presently available.

Human Huntingtin (hHtt) is a large 3144 aa protein. An important advance in the study of this protein was the discovery of the importance of its proteolysis in apoptosis [3]. Proteolytic cleavages by caspases and calpains occur in both wild-type and mutant Huntingtin, but the frequency of these cleavages increase with the length of the polyQ [3] [4] [5] [6] [1]. This leads to an abnormal accumulation of short toxic N-terminal polyQ-hHtt fragments in an HD context, driving inappropriate apoptosis [3] [7] [8]. These fragments are able to aggregate with themselves and other proteins, and form large cytoplasmic and nuclear inclusions [9]. The production of these polyQ-containing fragments has several patho-physiological consequences for the affected cells, which can include proteasome impairment, mitochondrial dysfunction and oxidative stress, excitotoxicity, transcriptional dysregulation or cell death. The ultimate accumulation of N-truncated forms of polyQ-hHtt in insoluble protein aggregates constitutes an important pathological hallmark of HD [10] [11] [7] [8] [12]. In post-mortem brains of HD patients, aggregates are enriched with N-terminal truncated forms of polyQ-hHtt [9]. Therefore, blocking mutant Huntingtin proteolysis was found to be beneficial in treating HD mice [7] [10] [11]. In general, HD models with short truncations of the expanded Htt protein present more severe phenotypes that also appear earlier than hHtt full-length expressing models [8] [13]. Mouse [14] and *Drosophila* HD models [13] that express full-length mutant Huntingtin do not present all of the different hallmarks of HD. For instance, flies expressing full-length mutant polyQ-hHtt only show synaptic dysfunction [13]. Furthermore, a selective neurodegeneration of the striatum and the cortex is observed in full-length mutant Htt mouse models, which is correlated with a selective nuclear localization of mutant Htt in these neurons [8] [11]. However, models expressing full-length mutant Htt do not display those defects that appear later in pathogenesis, such as widespread nuclear inclusions in all the brain, and massive neuronal dysfunction and cell death. These late defects are associated with the release and accumulation of polyQ-hHtt N-terminal truncated forms, which amplifies the aggregation process. A reduced cleavage efficiency of the full-length Htt might explain why animal models with full-length Htt only partially reproduce HD pathogenesis [15]. Therefore, important advances in the study of HD came from the development of transgenic models in mice or *Drosophila*, where only N-terminal portions of human polyQ-hHtt were expressed [16] [17] [18].

In *Drosophila*, transgenic models have been constructed that express N-terminal portions of human hHtt carrying different lengths of the polyQ domain (Figure S1). These cover either exon 1, corresponding to the first 67 amino acids (hHtt^67aa^) [16] [19], or longer N-terminal regions covering 171 aa (hHtt^171aa^) [20], or 548 aa (hHtt^548aa^) [21]. In the presence of expanded polyQ (>35), all of these constructs behave similarly by forming aggregates and leading (for example) to eye degeneration, regardless to the length of the N-terminal hHtt sequence (67 aa, 171 aa or 548 aa).

These models have been particularly helpful in drug screening [22]. However, until now only a few drugs have been tested in HD patients, with limited impacts. Indeed, given the multiple pathogenic mechanisms involved in this disease, it is expected that a compound that targets one pathological mechanism may not be effective alone. An attractive option for treating HD is of course to specifically target the mutant polyQ-hHtt, which is a strategy now actively pursued [23] [24] [6].

Apart from the dominant function of polyQ-hHtt, loss of function of the wild-type protein may also contribute to the disease pathogenesis [25] [26] [27] [28] [20]. In support of this hypothesis, we previously showed that the ratio between the wild-type hHtt N-terminal fragment and the mutant polyQ-hHtt was critical for the onset of polyQ-hHtt associated-defects, in a *Drosophila* model of HD [20].

The question that we have addressed here is what part of the wild-type Huntingtin is important for this protective effect. We first screened for peptides, derived from human Htt, that were able to prevent polyQ-hHtt aggregation in HeLa cells. This allowed the identification of a 23 aa protective peptide (P42), whose protective role was tested *in vivo* in flies. In this report we illustrate how P42 was not only able to prevent aggregation, but also clearly improved all tested polyQ-hHtt induced-defects, such as axonal trafficking of vesicles, larval locomotion, eye degeneration and adult survival. Our data also reveal that P42 was protective for HD models only, and not for other polyQ disease models. Altogether, our data suggest that P42 is acting on polyQ-hHtt by preventing both its aggregation and the ensuing devastative effects to the organism.

## Results

### Identification of Intrinsic Huntingtin Peptides that Prevent Polyq-hHtt Aggregation

**Figure 1:**
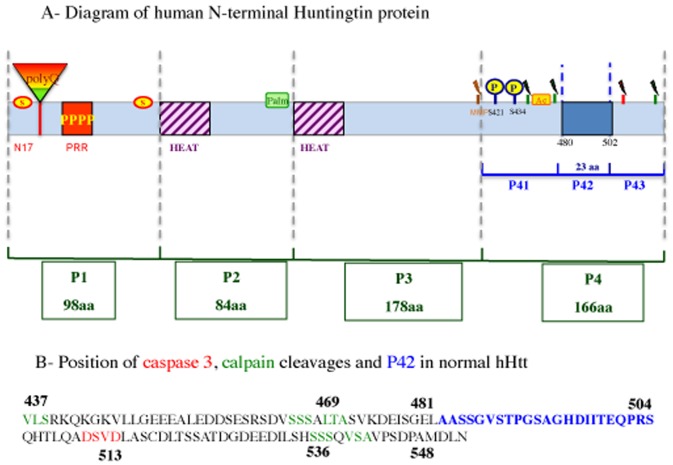
Schematic diagram of the 548 aa N-terminal part of human Huntingtin. A- The different domains of human Huntingtin protein (hHtt) are indicated: Polyglutamine tract (PolyQ), N17 and Proline rich (PRR) domains covering exon 1, as well as the HEAT repeats that share homologies with *Drosophila* Huntingtin; the sites of cleavage by caspase (in red), calpain (in green) or metallomatrixprotease (MMP); and posttranslational modifications, such as sumoylation (S), palmitoylation (palm), acetylation (Ac) and some of the phosphorylation (P) sites [49]. The positions and/or sizes of P1, P2, P3, P4, P41, P42 and P43 are indicated. Note that P1 is 98aa long, and does not contain any polyQ (0Q). B- The amino acid sequence of P42 is shown (in blue), as well as the caspase (in red) and calpain (in green) sites that surround P42 and that are otherwise indicated in A. Note that the aa position is numbered according to a protein containing 23Q.

**Figure 2:**
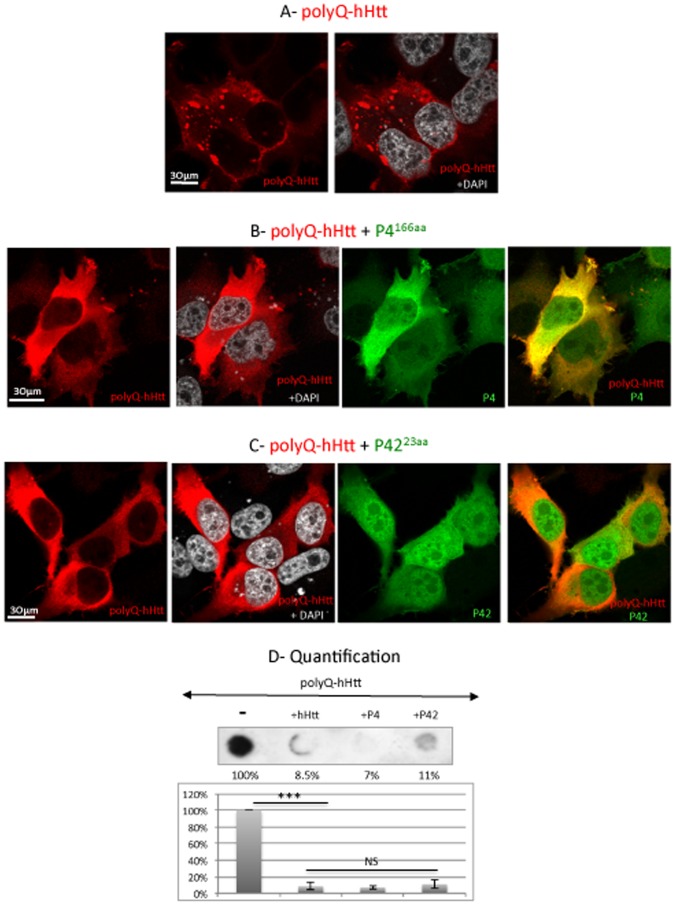
P42 reduces aggregate formation of polyQ-hHtt in HeLa cells. HeLa cells were transfected with GFP-138Q-hHtt^171aa^ (detected in red), in the presence of: A- empty vector; B- Cherry-P4^166aa^; or C- Cherry-P42^23aa^ expressing vectors detected by the Cherry tag (in green). DAPI staining is shown in grey. D- Quantification of aggregation by dot-blot filtration retardation assay on cells transfected with GFP-138Q-hHtt^171aa^, in the presence of empty vector (-), 0Q-hHtt^548aa^, P4^166aa^ or P42^23aa^ as indicated. Aggregates were detected with an anti-GFP antibody. Quantification was performed on 3 independent experiments and is reported on a graph as percentages with respect to the control (-) set up at 100%. Data were analyzed by using the *Student’s t-test*: ***p<0.001. The presence of hHtt covering either 548 aa, 166 aa or 23 aa leads to equivalent rescues that are not significantly different (NS).

In a previous work, we determined that overexpression of a 548 aa N-terminal part of wild-type human Htt (0Q-hHtt^548aa^) was able to prevent 138Q-hHtt^171aa^ aggregation in HeLa cells [20]. In order to identify the protective region of wild-type hHtt, we screened for peptides, within the human 0Q-hHtt^548aa^, that were able to prevent 138Q-hHtt^171aa^ aggregation in HeLa cells. Within the 548 aa protein sequence, four peptides were designed according to known protein domains, such as the HEAT repeats that share homologies with *Drosophila* Htt [29] [20] (Figure 1A). These peptides (referred to as P1, P2, P3 and P4) were tested in HeLa cells co-transfected with a 138Q-hHtt^171aa^ expressing construct (Figure S1). When transfected alone, 138Q-hHtt^171aa^ formed cytoplasmic aggregates as visualized by immunocytochemical detection (Figures 2A, S2). When each of the four designed peptides was co-transfected with 138Q-hHtt^171aa^, we found that only P4 (aa 382–548 of hHtt) was able to rescue polyQ-hHtt aggregation (Figure 2B), whereas the other peptides had no effect (data not shown).

Since our previous work showed that the N-terminal part of *Drosophila* Htt covering 620 aa also prevents 138Q-hHtt^171aa^ aggregation [20], we checked for conserved features present in both the 620 aa of *Drosophila* dHtt and the 166 aa of human P4 (aa 382-548 region of hHtt) (Figure S3). We thus identified a 23 aa region in the middle of P4 (aa 482-504; Figure 1B) that shares homologies between human and *Drosophila* Htt. Consequently, we further divided P4 into 3 fragments (P41, P42 and P43) with P42 corresponding to the 23 aa homology sequence. When these peptides were tested for their ability to rescue 138Q-hHtt^171aa^ aggregation in HeLa cells, only P42 was able to completely rescue this defect (Figures 2C), whereas the other peptides had no such effect (Figure S5 and data not show). These results showed that, based on immunocytochemical detection of aggregates, P42 was able to inhibit 138Q-hHtt^171aa^ aggregation in cultured human cells.

Inhibitory effects on aggregation were also quantified biochemically by filter retardation assays onto cellulose acetate membrane, in which polyQ-hHtt aggregates are specifically retained [30] (Figure 2D, and data not shown). Filter retardation assays indicated that the aggregation level falls from 100% (control) to 7% in the presence of P4, and to 11% in the presence of P42 (Figure 2D). This confirmed that the “AASSGVSTPGSAGHDIITEQPRS” 23 aa peptide (P42) was able to prevent 138Q-hHtt^171aa^ aggregation as efficiently as hHtt^548aa^ or P4^166aa^ (Figure 2D).

### Protective Effect of P42 Peptide *in vivo* on polyQ-hHtt Aggregation

**Figure 3:**
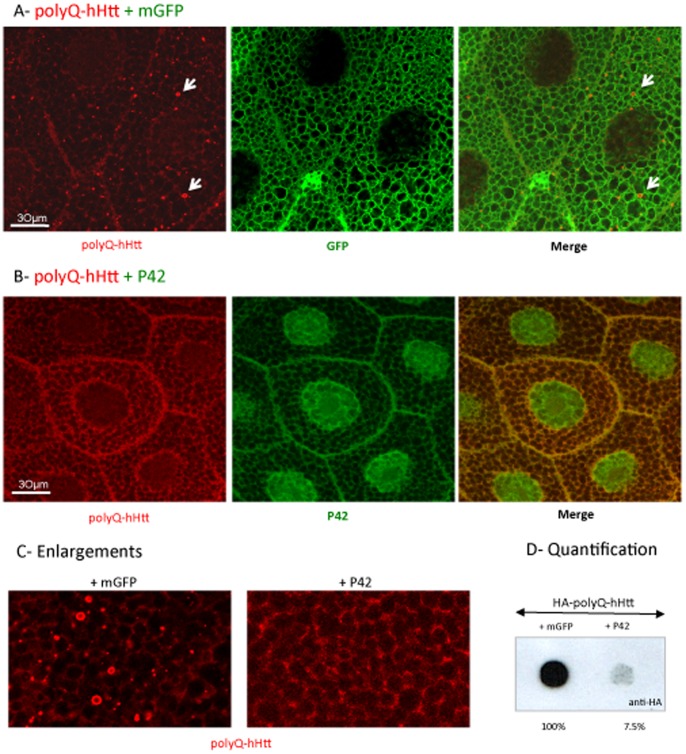
P42 reduces aggregate formation of polyQ-hHtt in salivary glands. A–C: Anti-HA detection (in red) of HA-138Q-hHtt^171aa^ expressed in salivary glands of *MS1096*-Gal4, UAS-HA-138Q-hHtt^171aa^/+ larva, in the presence of either A- one copy of a membrane-associated GFP neutral transgene (UAS-mGFP) (in green) or B- one copy of P42 (UAS-GFP-P42) (in green). Focal plan is at the level of the nuclei. Merged images are shown as indicated. A high magnification zoom from another focal plane is shown in C (HA staining only). D- Quantification of aggregation by dot-blot filtration assay performed on two independent sets of experiments; the percentage of aggregates formed in presence of P42 was determined with respect to the control (+mGFP) set up at 100%.

We further tested the ability of P42 to rescue polyQ-hHtt aggregation *in vivo* in a whole organism, using a *Drosophila* model of HD. For this, we constructed transgenic flies that express P42 (either GFP or Myc tagged in its N-terminal end) under the control of UAS sequences. The HD model used for this experiment consisted of transgenic flies expressing a HA-tagged polyQ-hHtt (HA-138Q-hHtt^171aa^), also under the control of UAS sequences [20] (Figure S1). Using the UAS/Gal4 system [31] with the Gal4 driver *MS1096*, P42 was first examined for its ability to prevent aggregation in non-neuronal cells such as salivary gland cells; these cells are particularly large, which facilitated visualizing the aggregates. Expression of 138Q-hHtt^171aa^ led to its aggregation (Figure 3A arrows, enlargement in C). It should be noted that this experiment was performed with transgenic flies also containing a neutral UAS transgene (UAS-mCD8-GFP) in order to homogenize the number of UAS constructs. This allowed for similar genetic conditions in all experiments, with the neutral UAS transgene being replaced by a UAS-P42 when needed. The 138Q-hHtt^171aa^ aggregates were essentially localized in the cytoplasm, as assessed by immunocytochemistry (Figure 3A). When the UAS-mCD8-GFP neutral transgene was replaced by UAS-GFP-P42, 138Q-hHtt^171aa^ aggregates were no longer detected (Figure 3B and quantification on Figure S4). In this case, 138Q-hHtt^171aa^ was visible as a diffuse cytoplasmic staining (Figures 3B, 3C), similar to normal Htt staining which overlaps the microtubule staining (Figure S6), as we demonstrated in a previous work [20]. Notably, P42 is detectable in both the nucleus and the cytoplasm, and the cytoplasmic fraction also shows the same profile than Htt (Figures 3B, S6). Even though P42 is slightly concentrated in the nucleus of salivary glands, as found in HeLa cells (Figure 2C), its expression does not disturb the nucleo-cytoplasmic distribution of wild-type hHtt (Figure S6). This indicates that P42 was able to prevent polyQ-hHtt aggregation, and was able to restore a normal cytoplasmic repartition of Huntingtin, along the ß-tubulin network (Figure S6). Quantification of aggregates by filter retardation assays confirmed that 138Q-hHtt^171aa^ aggregation in salivary glands was prevented by P42 (Figure 3D), which corroborates the results obtained in HeLa cells.

In conclusion, these data show that P42 is able to prevent polyQ-hHtt aggregation both in cultured human cells and *in vivo* in a whole organism.

### Protective Effect of P42 Peptide on Impaired Axonal Transport Induced by polyQ-hHtt

We then studied *in vivo*, in *Drosophila*, the protective effect of P42 on polyQ-hHtt induced neuronal phenotypes that are distinct from protein aggregation alone. We first focused on axonal transport that is impaired by the presence of mutant polyQ-hHtt [32] [33]. Indeed, a defect in axonal transport could be one cause for the decrease in neurotrophic factor delivery (such as BDNF) and would participate in the selective striatal degeneration that occurs in HD patients [26].

Our axonal transport study focused on vesicular transport of GFP-tagged neuropeptide Y (NPY-GFP) within the *Drosophila* larval motoneurons, using an *OK6*-Gal4 driver [34] [35]. Neuropeptide-Y is a fast axonal transport cargo that is diffusely distributed in wild-type nerves, but accumulates when transport is compromised; forming vesicle clogs along the axons.

**Figure 4:**
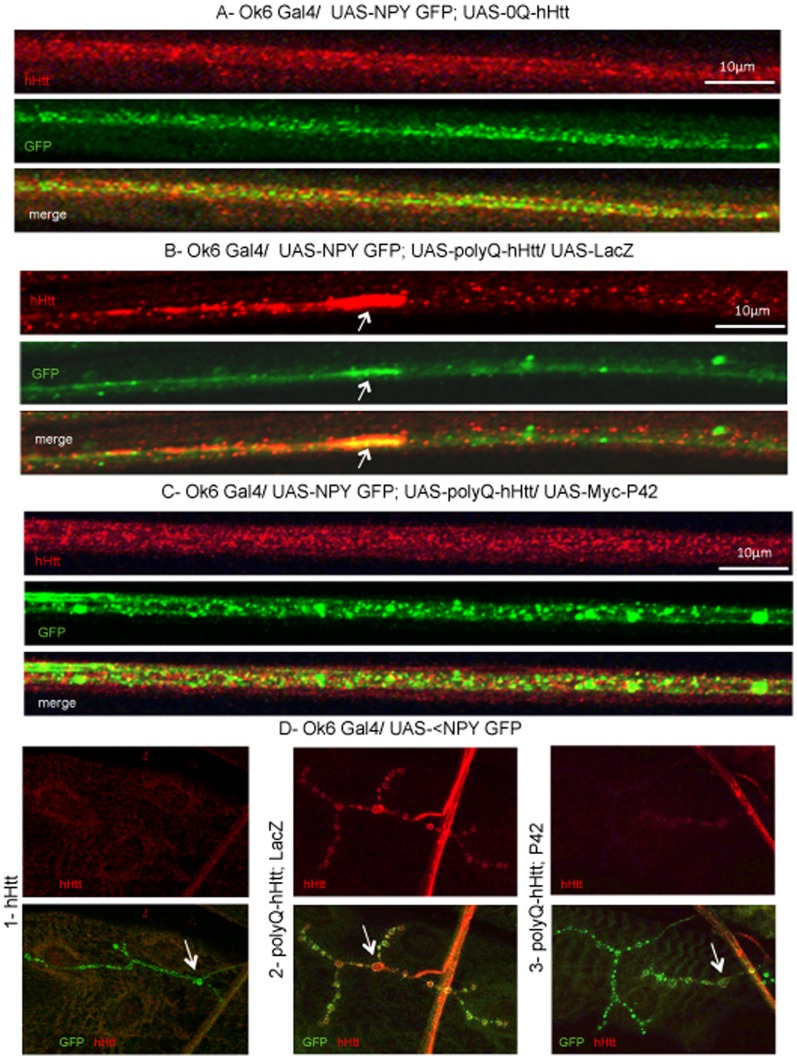
Distribution of neuropeptide Y-GFP vesicles along the axons and at the neuromuscular junctions (NMJ) of *Drosophila* larvae. Vesicular neuropeptide-Y-GFP (NPY-GFP) expression was visualized by anti-GFP immunostaining (in green) in larval motoneurons expressing hHtt^548aa^ detected by anti-hHtt (Hu-4C8) (in red), in different genotypes: A- *OK6*-Gal4/+; UAS-NPY-GFP/UAS-0Q-hHtt^548aa^ (hHtt). B- *OK6*-Gal4/UAS-128Q-hHtt^548aa^; UAS-NPY-GFP;/UAS-LacZ (polyQ-hHtt; LacZ). C- *OK6*-Gal4/UAS-128Q-hHtt^548aa^; UAS-NPY-GFP/UAS-P42 (polyQ-hHtt; P42). Merged images are shown. D- Muscle 4 NMJs are shown in the different genetic backgrounds: 1- *OK6*-Gal4/+; UAS-NPY-GFP/UAS-0Q-hHtt^548aa^ (hHtt). 2- *OK6*-Gal4/UAS-128Q-hHtt^548aa^; UAS-NPY-GFP;/UAS-LacZ (polyQ-hHtt; LacZ). 3- *OK6*-Gal4/UAS-128Q-hHtt^548aa^; UAS-NPY-GFP/UAS-P42 (polyQ-hHtt; P42). When expressing wild-type hHtt, NPY-GFP and 0Q-hHtt^548aa^ are uniformly distributed along the axons and 0Q-hHtt^548aa^ did not reach the NMJ boutons whereas NPY-GFP did (arrows in D1). In contrast to this control, 128Q-hHtt^548aa^ is found in large aggregates (see arrows in B and Figure S7) and NPY-GFP vesicles form clogs along the axons, and 128Q-hHtt^548aa^ accumulates at the NMJ boutons (arrows in D2). Note that the presence of P42 restores the normal distribution of NPY-GFP and 128Q-hHtt^548aa^ both along the axons and at the NMJ boutons (arrows in D3).

**Figure 5:**
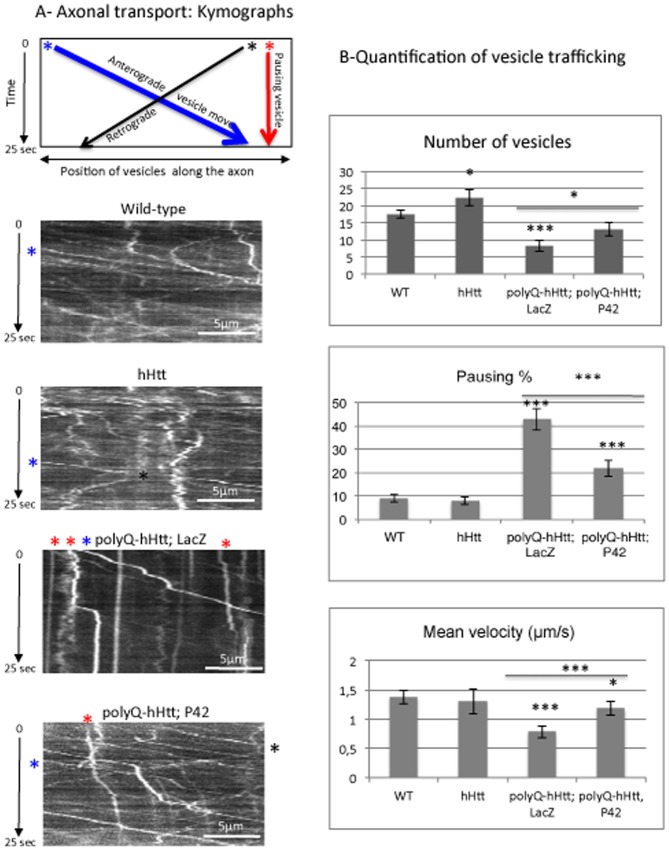
Axonal transport of neuropeptide Y-GFP vesicles. A- A representative schematic kymograph of vesicular movement along the axons is shown. The data were obtained for vesicles going in anterograde (* in blue) or retrograde (* in black) directions, as well as for vesicles that do not move (* in red). The starting point of vesicles is shown as examples and follow a trajectory according to the color code. Kymographs of vesicular movement are shown for four genotypes: Wild-type, *OK6*-Gal4/+; UAS-NPY-GFP/UAS-0Q-hHtt^548aa^ (hHtt), *OK6*-Gal4/UAS-128Q-hHtt^548aa^; UAS-NPY-GFP/UAS-LacZ (polyQ-hHtt; LacZ), and *OK6*-Gal4/UAS-128Q-hHtt^548aa^; UAS-NPY-GFP/UAS-P42 (polyQ-hHtt; P42). Note that only few vesicles are detectable in (polyQ-hHtt; LacZ) larvae and most of them are pausing (* in red), which is not the case in presence of P42. B- Quantification of different parameters (number of vesicles, % of pausing and mean velocity of vesicles) for NPY-GFP vesicular movement was performed in the genetic backgrounds described in A (*n* = 9-13 larvae). Mean values are compared to wild-type conditions (WT), or to polyQ-hHtt; LacZ. Data were analyzed with the *Student’s t-test* (*p<0.05, ***p<0.001). As compared to the control (WT), the presence of 128Q-hHtt^548aa^ reduced vesicle number, increased the percentage of pausing, and reduced the mean velocity. Note that the presence of P42 rescued the defects induced by 128Q-hHtt^548aa^.

We first analyzed the global distribution of the NPY-GFP vesicles on fixed larvae with different genetic conditions (Figure 4). When expressed in motoneurons, the wild-type 0Q-hHtt^548aa^ protein was found all along the axons, which corroborates previous studies that showed Htt particles along the microtubules [36] [37]. Interestingly, we observed that high expression of 0Q-hHtt^548aa^ did not induce clogs of NPY-GFP vesicles (Figure 4A). On the contrary, mutant 128Q-hHtt^548aa^ formed large aggregates along the axons (Figures 4B, S7). In addition, it induced the accumulation of NPY-GFP vesicles within clogs, especially at the location of the polyQ-hHtt aggregates [21] (Figure 4B arrows). This was performed in the presence of a control UAS-LacZ transgene, to ensure a similar number of UAS constructs under all tested conditions. When this control transgene was replaced by UAS-P42 to induce the expression of P42, we found that 128Q-hHtt^548aa^ formed fewer aggregates (Figure 4C and quantification on Figure S7). In addition, trafficking of NPY-GFP vesicles along the axons was recovered, with fewer vesicular clogs (Figure 4C) and more individual, potentially moving, vesicles (Figure 4C, and quantifications on Figure 5B).

We also analyzed the *Drosophila* larval neuromuscular junction (NMJ), a glutamatergic synapse that is largely studied both developmentally and physiologically [38] (Figure 4D). When wild-type 0Q-hHtt^548aa^ protein was expressed in motoneurons it was barely detectable at the synapse (Figure 4D1), although it was visible within the nerves (Figure 4A). On the contrary, expression of 128Q-hHtt^548aa^ protein in motoneurons resulted in clear hHtt immunoreactivity in the nerves (Figure 4B) but also at the NMJ (Figure 4D2), indicating that the extended polyQ domain led to the accumulation of hHtt at the NMJ (Figure S8). The accumulation of 128Q-hHtt^548aa^ did not have an obvious effect on the presence of NPY-GFP vesicles at the NMJ (Figure 4D2). As similarly observed for the aggregation phenotype, co-expression of P42 with the 128Q-hHtt^548aa^ protein eliminated the accumulation of Huntingtin at the NMJ (Figure 4D3).

Altogether, these results show that P42, in addition to its rescuing effect on 128Q-hHtt^548aa^ aggregates, is also able to rescue polyQ-hHtt mislocalization at the NMJ, as well as polyQ-hHtt-induced axonal vesicular clogs.

We next investigated in further detail the dynamic vesicle movement along the axons, using a non-invasive *in vivo* technique to follow the vesicle trajectories, through the cuticle of living larvae [34] [35]. Vesicle motion in motoneuron axons was captured in short movies and was recapitulated in representative kymographs for each genotype (Figure 5A). Vesicle counting and tracking allow the investigation of a range of characteristic parameters, including the number of vesicles, the proportion of vesicles that are pausing, and the mean velocity of mobile vesicles (Figure 5B). We first quantified the number of isolated, potentially moving vesicles within a fixed nerve length, for the first frame of each movie. In the control condition, a large number of NPY-GFP vesicles were traveling in motoneurons in both retrograde and anterograde directions (Figure 5A). The number of traveling vesicles was significantly enhanced in the presence of wild-type 0Q-hHtt^548aa^, probably because of its effect on microtubule-based transport, as previously described [37] [26] (Figures 5A, 5B). Expression of wild-type 0Q-hHtt^548aa^ did not modify either the mean pausing time or the mean velocity of these traveling vesicles (Figure 5B). On the contrary, there were fewer vesicles in the presence of 128Q-hHtt^548aa^, and existing vesicles showed an increased pausing rate (Figures 5A, 5B), as well as a decreased mean velocity (Figure 5B).

When co-expressed with 128Q-hHtt^548aa^, P42 induced a rescue in the number of vesicles within the nerve to the level of wild-type larvae. In addition, P42 significantly rescued the dynamic vesicle movement affected by the presence of the mutant 128Q-hHtt^548aa^ protein, by reducing the proportion of pausing vesicles and enhancing their mean velocity. Note, however, that this rescue was not complete and did not reach the wild-type level (Figures 5A, 5B).

In conclusion, we show that P42 is able to improve the vesicular trafficking defects induced by the mutant polyQ-hHtt within axons, by restoring the global number and motion of vesicles.

### Protective Effect of P42 on Physiological Behaviors Affected by polyQ-hHtt

Next, we analyzed the protective effect of P42 on 128Q-hHtt^548aa^ induced defects at a more integrated level, focusing on larval locomotion and adult lifespan.

**Figure 6:**
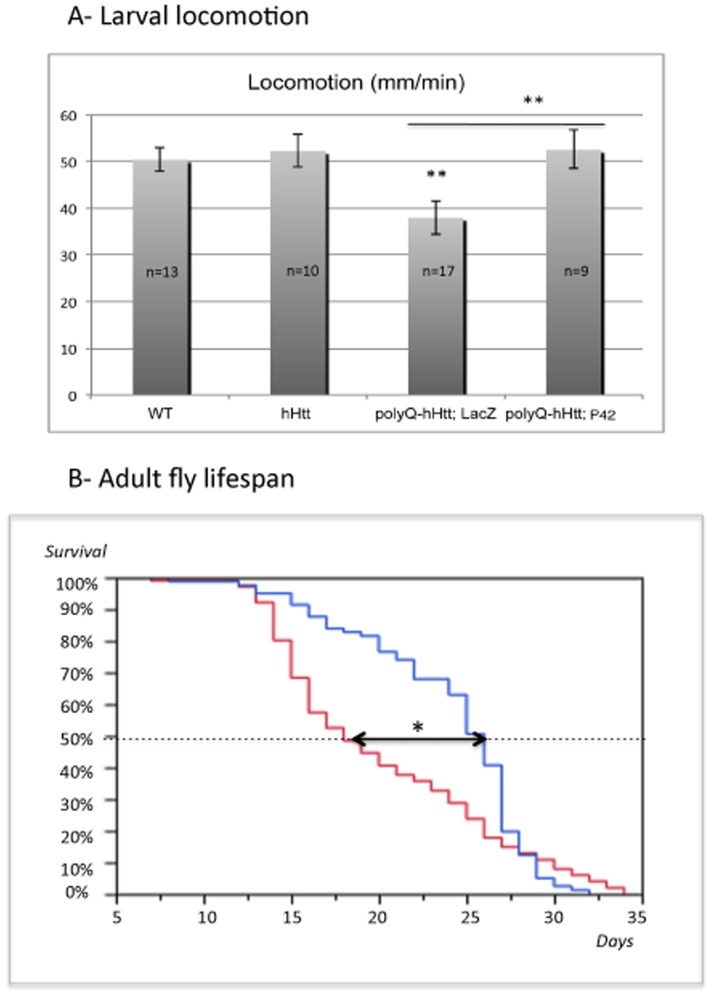
P42 rescues physiological behaviors induced by polyQ-hHtt. A- Larval locomotion was examined during 2 min in four different genetic backgrounds: Wild-type (WT), *OK6*-Gal4/+; UAS-NPY-GFP/UAS-0Q-hHtt^548aa^ (hHtt), *OK6*-Gal4/UAS-128Q-hHtt^548aa^; UAS-NPY-GFP/UAS-LacZ (polyQ-hHtt; LacZ), and *OK6*-Gal4/UAS-128Q-hHtt^548aa^; UAS-NPY-GFP/UAS-P42 (polyQ-hHtt; P42). Mean values are calculated on (*n* = 9 to 17), as indicated. Data were analyzed with the *Student’s t-test* (** *p<0.01*). Compared to the WT control, the presence of 128Q-hHtt^548aa^ reduced larval locomotion, whereas P42 rescued the locomotion defect. B- Analysis of adult lifespan for UAS-128Q-hHtt^548aa^ *; elav*-Gal4/UAS-LacZ (polyQ-hHtt; LacZ in blue) and UAS-128Q-hHtt^548aa^ *; elav*-Gal4/UAS-P42 (polyQ-hHtt; P42 in red) flies are shown. The number of flies that survived from each cohort was evaluated once per day. Mean, median and maximum survival times (in days) were calculated from survival curves by using the Kaplan-Meier analysis; percentage of survival is provided. The arrow indicates the difference in age at which 50% of the flies have died; the median survival is increased in the presence of P42. *P* value was calculated by using log rank statistics (*p = 0.0148).

We analyzed the locomotion of larvae expressing wild-type or mutant 128Q-hHtt^548aa^ in motoneurons, using the *OK6*-Gal4 driver (Figure 6A). The presence of wild-type 0Q-hHtt^548aa^ protein did not affect larval locomotion (mean of 52 mm/min) (Figure 6A). On the contrary, expression of 128Q-hHtt^548aa^ protein significantly impaired larval locomotion with a 25% speed reduction, in accordance with the presence of axonal transport defects. Remarkably, co-expression of P42 completely rescued this locomotor defect.

It has previously been reported that expression of polyQ-hHtt in all *Drosophila* neuronal cells reduces fly longevity [39] [40]. Therefore, we next examined the rescuing effect of P42 on adult fly viability (Figure 6B). To investigate this, we performed survival curves for different cohorts of flies expressing polyQ-hHtt (128Q-hHtt^548aa^) in all neurons (under control of the *elav*-Gal4 driver), either in the absence or presence of P42. The normal lifespan of wild-type flies ranges around 60 days [39] [40]. Expression of P42 alone did not alter the normal viability of the flies (data not shown), suggesting that its overexpression is not toxic. 128Q-hHtt^548aa^ adults displayed a maximum lifespan of 33 days with a median lifespan (the age at which 50% of the flies have died) occurring at 18 days (Figure 6B). The maximal survival time did not change when P42 was co-expressed, although the median adult lifespan increased to 26 days, which corresponds to a 24% increase (Figure 6B). The mean viability of the flies was also significantly enhanced in the presence of P42. From these results, we can conclude that P42 significantly improves the survival of the HD flies, without enhancing their maximum lifespan. This indicates a partial rescue of P42 on longevity defects induced by mutant polyQ-hHtt.

To conclude, we found that P42 presents protective properties for a range of polyQ-hHtt induced phenotypes, from cellular defects to behavior.

### Protective and Specific Effect of P42 on Cellular Death

Having confirmed the rescuing effect of P42 for a range of phenotypes, we finally focused on the most studied “degeneration” phenotype in the fly model, *i.e*. cell death in the eye. Because this phenotype is also common to many fly models of neurodegenerative diseases, this assay allowed us to examine whether P42 could additionally rescue neurodegeneration associated with other polyQ mutant proteins, such as Ataxins.

**Figure 7:**
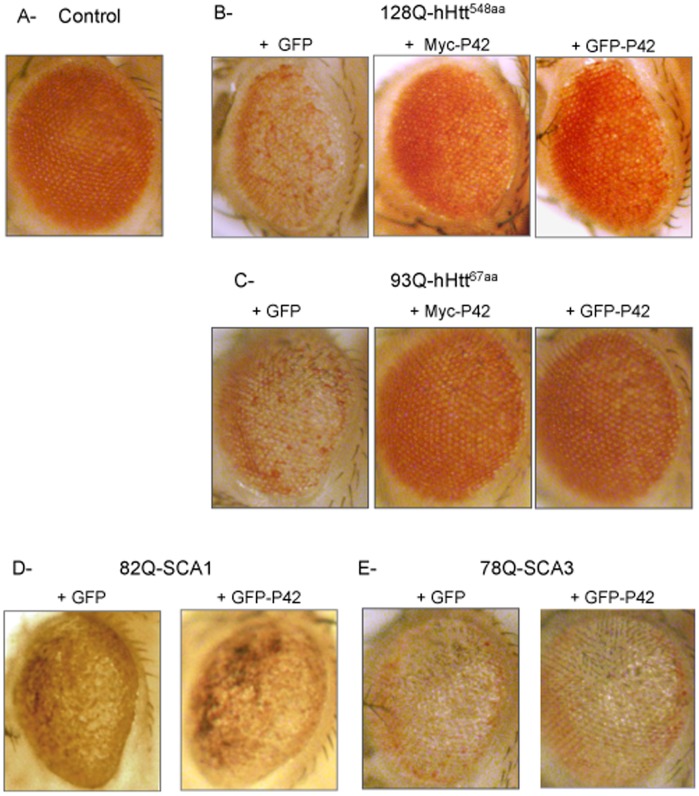
Influence of P42 on eye toxicity induced by different polyQ mutant proteins. A- As a control to examine eye toxicity, we used *GMR*-Gal4/UAS-GFP flies that exhibit normal eyes. Adult eye phenotypes were analyzed in four different genetic backgrounds: B- *GMR*-Gal4/UAS-128Q-hHtt^548aa^, C- *GMR*-Gal4/+; UAS-93Q-hHtt^67aa^/+, D- *GMR*-Gal4/+; UAS-82Q-SCA1/+, and E- *GMR*-Gal4/+; UAS-78Q-SCA3/+, which was tested either in the presence of the UAS-GFP neutral transgene, or in the presence of P42 tagged with Myc or GFP, as indicated. PolyQ-hHtt flies carrying the UAS-GFP neutral transgene exhibited eye degeneration, with depigmented ommatidia. On the contrary, polyQ-hHtt flies exhibited normal eyes in presence of P42, regardless if this was tagged with Myc or GFP (in B and C). Note that other models of polyQ-induced ataxias exhibited eye degeneration in the presence of either a neutral UAS-GFP transgene or the UAS-P42 transgene (in D and E), indicating that the P42 rescue effect is specific to polyQ-hHtt.

PolyQ-hHtt expression with the *GMR*-Gal4 driver in developing eyes leads to adults showing a loss of eye pigmentation, associated with abnormal ommatidial arrays (as detected by scanning electron microscopy) and a loss of photoreceptors [19] [21] [20] (compare Figure 7A to 7B or 7C and data not shown). Here, we used different polyQ-hHtt transgenes that encode different truncated forms of polyQ-hHtt (Figures S1, 7B, 7C). We first analyzed the rescuing activity of P42 on eye toxicity generated in flies expressing either 128Q-hHtt^548aa^ [21], (containing the P42 sequence; Figures 7B, S1) or a short 93Q-hHtt^67aa^ [19] that does not include the P42 sequence (Figures 7C, S1). Interestingly, the eye toxicity induced by both transgenes was rescued in the presence of P42 tagged with either Myc or GFP (Figures 7B, 7C). This indicates that P42 is able to rescue polyQ-hHtt toxicity induced by different forms of polyQ-hHtt N-terminal fragments. In particular, P42 can rescue eye toxicity induced by a polyQ-hHtt transgene containing only the first 67 aa (exon 1) of human Huntingtin (Figure 7C).

We then wondered whether P42 could also rescue *Drosophila* models of other polyQ neurodegenerative diseases, such as spinocerebellar ataxia type 1 (82Q-SCA1) [41] (Figure 7D) or spinocerebellar ataxia type 3 (78Q-SCA3) [42] (Figure 7E). Interestingly, P42 was not able to rescue the eye degeneration induced by these other polyQ mutant proteins (Figures 7D, 7E).

This indicates that P42 has a protective effect that is specific to polyQ-hHtt induced toxicity.

## Discussion

This study is a continuation of our previous observation that wild-type Htt (human Htt^548aa^ and *Drosophila* Htt^620aa^) plays a protective role with respect to polyQ-hHtt induced toxicity [20]. We divided the human Htt^548aa^ into four peptides and tested each one for their protective effect. We initially expected P1, P2 or P3 to play such a role because they contain important protein domains. Indeed, P1 contains the N17 and PRR domains known to influence the aggregation process [43] [44]. P2 and P3 contain HEAT domains that are known to participate in protein-protein interactions [45] and that are also found in *Drosophila* Htt. Surprisingly, we determined that only P4 was able to efficiently block 138Q-hHtt^171aa^ aggregation in HeLa cells. Even though it does not contain any predicted domain, this 166 aa P4 peptide is particularly rich in proteolytic and phosphorylation sites that all impact the cleavage of the protein that plays important functions in the neurodegeneration process [5] [46] [47] [48] [49] [50]. We then searched for homologies between the 620 aa N-terminal part of *Drosophila* Htt and Human P4. This led to the identification of conserved features extending over a 23 aa peptide (P42) in the Human counterpart. A further partition of P4 revealed that indeed only P42 within P4 had efficiently retained the protective properties of the larger fragments (P4 or hHtt^548aa^). Whereas both P4 and P42 inhibit polyQ-hHtt aggregation, we could observe that their nucleo-cytoplasmic repartition is slightly different. They are both localized in the cytoplasm, which is in line with their role in prohibiting the formation of aggregates that are essentially detected in the cytoplasm of both culture cells and flies. However, P42 is also highly concentrated in the nucleus (Figures 2 and 3). As it has been demonstrated that nuclear inclusions of polyQ-hHtt appear in late stages of the disease [8] [11], the nuclear localization of P42 suggests that this peptide may also be protective for nuclear inclusions.

In flies, P42 was able to prevent aggregation induced by polyQ-hHtt, as it does in HeLa cells. Since the exact role of aggregates and their involvement in cell toxicity remain controversial [51], it was important to evaluate the impact of P42 on other cellular and functional phenotypes. When looking at polyQ-hHtt induced cellular defects, we observed that P42 was able to completely rescue some defects (including aggregation in salivary glands and polyQ-hHtt mislocalization at the NMJ), while others were only partially rescued (such as for instance the number of travelling vesicles along the axons). This incomplete recovery could result from the ability of soluble polyQ-hHtt to reduce kinesin-based axonal transport [33]. Thus, even when it is not involved in aggregates, the presence of soluble polyQ-hHtt might still affect axonal function. In accordance with our results at the cellular level, we could obtain either a full rescue (eye phenotypes, larval locomotion) or only a partial one (fly viability) at the physiological/behavioral level. Toxicity has been shown to be likely associated with both soluble and aggregated polyQ-hHtt levels [15]. Whereas aggregates are not formed in the presence of P42, the level of soluble polyQ-hHtt might be enhanced, which could explain the incomplete recovery. One way to improve these partial rescues obtained with P42, when considering therapeutic applications, could be to enhance the clearance of polyQ-hHtt through P42 targeting. To pursue this goal, fusion molecules comprising P42 and peptides required in macroautophagy [52] or in proteasomal degradation [53] could be engineered. Such processes have been shown to ameliorate the formation of aggregates in culture cells [53], as well as symptoms in mouse HD models [52] [54]. To ensure the safety of such a reagent, an important preliminary issue will be to verify that P42 preferentially binds polyQ-hHtt, and not the wild-type. Another way to better prevent the toxicity of polyQ-hHtt expression could be a combinatorial approach as already described [55], using the concomitant use of P42 and other potential therapeutic agents such as the QBP1 peptide that directly interferes with polyQ [40].

To explain P42 protective effect, we first excluded that P42 affects polyQ-hHtt level of expression both in cells (Figure S9), and *in vivo* in salivary glands (Figure S6). Hence, for its protective effect, P42 could act either indirectly by titrating proteins that are involved with the polyQ-hHtt in the disease, or more directly by interacting with the polyQ-hHtt. The fact that P42 was protective for polyQ-hHtt but not for other polyQ toxic proteins such as polyQ-SCA1 and polyQ-SCA3, and that it was able to rescue eye phenotypes induced by 93Q-hHtt^67aa^ (Figure 7) strongly suggest that if direct, P42 interaction might not occur through the polyQ, but must occur with the first 67 aa of Htt (exon 1). It is interesting to notice that exon1 contains the N17, and the PRR (Proline rich region) domains (Figure 1), both of which play a role in polyQ-hHtt aggregation, although through different mechanisms [44] [56] [57]. In particular, aggregation process requires a nucleation step, involving the self-interaction of the N17 domains. During this nucleation event, the polyQ-hHtt N-terminus adopts a structure that is able to associate with itself, which leads to a local concentration and the oligomerization of the mutant hHtt. The presence of the N17 domain was shown to accelerate the aggregation process [56]. As a result, factors (such as intrabodies for instance) targeting the N17 domain are able to suppress aggregation and the associated toxicity by inhibiting this nucleation step [58] [39] [46] [59] [60]. Therefore, by targeting exon1, P42 might interfere on both the nucleation and aggregation processes, leading, as observed, to a complete disappearance of polyQ-hHtt aggregates.

We can also notice that P42, which is a part of the hHtt protein, is localized within a region rich in cleavage sites. One hypothesis is that an endogenous role of P42 could be to slow Htt cleavage. Therefore, blocking mutant Huntingtin proteolysis by P42 could be protective, which is known to be beneficial in treating HD mice [7] [10] [11].

In this report, we identified a short peptide that is protective *in vivo* of polyQ-hHtt induced defects. The identification of this 23 aa peptide endogenous to hHtt that saves neuronal defects induced by polyQ-hHtt in flies opens a new avenue for future therapeutic strategies. In accordance to a clear therapeutic potential for this peptide, the use of P42 combines on the one hand, a clear specificity of action towards hHtt and on the other hand, the advantages of peptide technologies such as low toxicity, mainly because it is a part of the Huntingtin protein.

## Materials and Methods

### Drosophila Stocks

Stocks *MS1096*-Gal4 (on X) [61]; OK6-Gal4 (on II) [62]; UAS-SCA1-82Q [41]; UAS-SCA3-78Q [42]; *GMR*-Gal4 (on II); *elav*-Gal4 (on III); UAS-GFP (on II); UAS-mCD8-GFP (on III); and UAS-LacZ (on III) were obtained from the Bloomington *Drosophila* stock center. UAS-NPY-GFP was obtained from I. Robinson (Cambridge, U.K.). For *Drosophila* models of Huntington’s disease, we used the following stocks: UAS-93Q-Htt^67aa^, obtained from Leslie Thompson [19]; UAS-HA-138Q-hHtt^171aa^ [20], or UAS-128Q-Htt^548aa^, obtained from J. Troy Littleton [21]. UAS-0Q-Htt^548aa^ [21] was used for normal human Huntingtin. Note that only hHtt^171aa^ transgene is HA tagged at its N-terminal end. Since there is a direct link between polyQ-hHtt induced phenotypes and its level of expression, controls were always tested in the presence of a neutral UAS-transgene (UAS-LacZ or UAS-GFP or UAS-mCD8- GFP as indicated).

### Antibodies

Primary antibodies used include: anti-HA antibody (SC805 rabbit polyclonal; Santa Cruz), anti-HA (3F10 rat monoclonal; Roche), anti-Huntingtin (Hu-4C8 mouse monoclonal; Chemicon), anti-GFP antibody (polyclonal rabbit; Invitrogen), and anti-DsRED antibody (polyclonal rabbit; Clontech). Secondary antibodies (from Jackson) include: anti-rabbit antibody coupled with HRP or with Alexa488, and anti-mouse Cy3-conjugated antibody.

### Plasmid Constructions using Gateway

- pDon-P42 made by PCR on Human hHtt with two primers.

P42 up:GGGGACAAGTTTGTACAAAAAAGCAGGCTTCgctgcttcttcaggggtt, and P42 down: acagaacagccacggtcaGACCCAGCTTTCTTGTACAAAGTGGTCCCC.

PCR were recombined in Gateway destination vectors:

- The Cherry-TAG pCMV Gateway vector was provided by the MGC (Montpellier Genetic Collections).

- GFP-TAG is pDEST53 Gateway destination vector (Invitrogen).

- GFP-TAG; HA-TAG or MYC-TAG in pUASt gateway vectors were provided by the DGRC to construct *Drosophila* transgenic lines.

- Constructs were normally tagged at their 5′end.

### Transfection of Cultured HeLa Cells

- Cells were transfected with 1.5 µg total DNA using JetPei reagent (Qbiogene) for 20 hrs. Cells were rinsed with 1x PBS and fixed with 4% PFA for immunocytochemistry.

### Filter Retardation Assays

Protein extracts from L3 larval salivary glands or from HeLa cells were performed according to Mugat *et al.* (2008) [20]. Using a Bio-Rad dot-blot filtration unit, denatured protein samples were filtered through a cellulose acetate membrane (0.2 µm pore size, Schleicher & Schuell) that only retains aggregates. Membranes were then immunodetected with primary antibodies: rabbit polyclonal anti-HA antibody (1∶100), or rabbit polyclonal anti-GFP antibody (1∶5000;), as indicated. Subsequently, the membranes were incubated with the secondary antibody, anti-rabbit coupled HRP (1∶50,000). Immunoreactive spots were detected with chemiluminescent ECL substrate (Roche), and quantifications were performed using the ImageJ software.

### Construction of *Drosophila* Transgenic Lines

UAS-Myc-P42 and UAS-GFP-P42 were cloned in the Gateway DGRC vector and injected into embryos (BestGene Inc.).

### Immunostainings

Salivary glands of third instar male larvae were dissected and immunostained according to Mugat *et al.* (2008) [20]. Motoneurons and NMJs of third instar female larvae were dissected and immunostained according to Franco *et al.* (2004) [63]. Images were acquired by confocal microscopy.

### Non-invasive Tracking of Vesicles in Segmental Nerves of Living Larvae

Following the protocol of Talmat-Amar *et al.* (2011) [35], we anaesthetized third instar female larvae expressing NPY-GFP in their motor neurons with ether during 2 min. Living larvae were mounted ventral side up on a slide in 1% agarose with a coverslip, and viewed with a fluorescent microscope to track vesicle motion in nerves between segments A2–A3. 100-frame movies were recorded every 280 ms, for one nerve *per* larva (*n = *9–13). To quantify vesicle number, the total number of vesicles was counted along a distance of 21 µm in the first frame of each movie. For motion analysis, 20 vesicles were randomly selected in the first frame of each movie and tracked by ImageJ (using the plugin “manual vesicle tracking”). Vesicles with a velocity inferior to 0.01 µm/s were counted as pausing. Kymographs that permit visualizing the movement of all vesicles within a selected axonal segment *versus* time were created with imageJ (using the plugin “kymo”). The plugins were developed by F. Cordelières (Institut Curie, Orsay, France).

### Larval Locomotion Analysis

Wandering third-instar female larvae were placed in the middle of a 13.5 cm wide Petri-dish filled with grape juice agar (classically used for *Drosophila* egg collection). A grid pattern consisting of 0.5 cm^2^ squares was placed underneath the plate, and the number of sides crossed by each larva during 2 minutes was recorded. We averaged the values obtained for all larvae of each genotype.

### Eye Phenotype Analysis

We analyzed the eye degeneration and depigmentation in adult females where UAS transgene expression was induced by the *GMR*-Gal4 driver. Phenotypes were analyzed on 10-day old UAS-93Q-hHtt^67aa^ flies that had been transferred to 29°C at L3; 15-day old UAS-128Q-hHtt^548aa^ flies raised at 29°C; 2-day old UAS-SCA1-82Q flies that were raised at 29°C since L3; and 2day old UAS-SCA3-78Q flies raised at 25°C.

### Longevity Assay

UAS-128Q-hHtt^548aa^/*elav*-Gal4 (III) flies were analyzed for mean and median survival in the presence of either LacZ (control) or GFP tagged P42. The cohorts were tested for each genotype (101 and 81 males, respectively), which were collected within 24 hrs after eclosion and maintained on food at 25°C. Survival of flies was evaluated every day.

## Supporting Information

Figure S1 **Diagram of N-truncated parts of human Huntingtin proteins expressed in the different Drosophila models used in this report.**
(PDF)

Figure S2 **Low magnification images of HeLa cells transfected with polyQ-hHtt.**
(PDF)

Figure S3 **Alignment between dHtt (1-620aa) and hHtt P4 (166aa).**
(PDF)

Figure S4 **Quantification of aggregates, using ImageJ software on confocal stacks.**
(PDF)

Figure S5 **PolyQ-hHtt expression in HeLa cells.**
(PDF)

Figure S6 **In salivary glands, hHtt intensity and pattern does not change in presence of P42.**
(PDF)

Figure S7 **Confocal images of third larval peripheral nerves expressing hHtt^548aa^.**
(PDF)

Figure S8 **Quantification of HHtt immunoreactivity at the NMJ.**
(PDF)

Figure S9 **Protein extracts from HeLa cells transfected by GFP-polyQ-hHtt.**
(PDF)
