# Supplementary material for: A Huntingtin Peptide Inhibits PolyQ-Huntingtin Associated Defects
Source: PLoS One. 2013 Jul 4;8(7):e68775. doi: 10.1371/journal.pone.0068775 (PMC3701666; doi:10.1371/journal.pone.0068775)
Supplement: Figure S1 — Diagram of N-truncated parts of human Huntingtin proteins expressed in the different Drosophila models used in this report. (PDF) [file pone.0068775.s001.pdf]

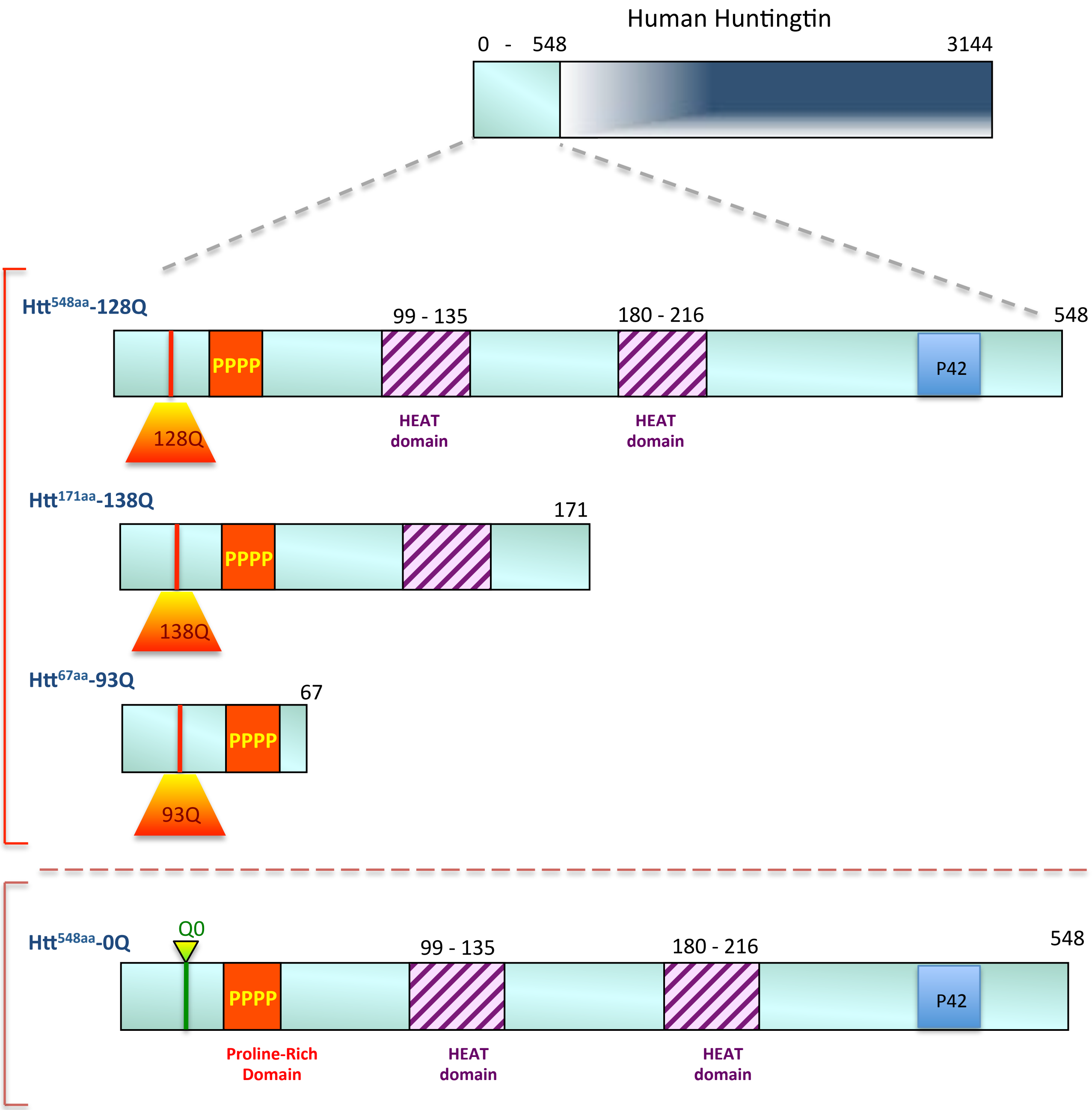

Figure S1: Diagram of N-truncated parts of human Huntingtin proteins expressed in the different *Drosophila* models used in this report. Note that only the $hHtt^{171aa}$ construct contains an HA-Tag in its N-terminus (Mugat et al., 2008). The $hHtt^{171aa}$ construct used in HeLa cells is GFP-tagged at its N-terminus.
