# Supplementary material for: A Huntingtin Peptide Inhibits PolyQ-Huntingtin Associated Defects
Source: PLoS One. 2013 Jul 4;8(7):e68775. doi: 10.1371/journal.pone.0068775 (PMC3701666; doi:10.1371/journal.pone.0068775)
Supplement: Figure S2 — Low magnification images of HeLa cells transfected with polyQ-hHtt. (PDF) [file pone.0068775.s002.pdf]

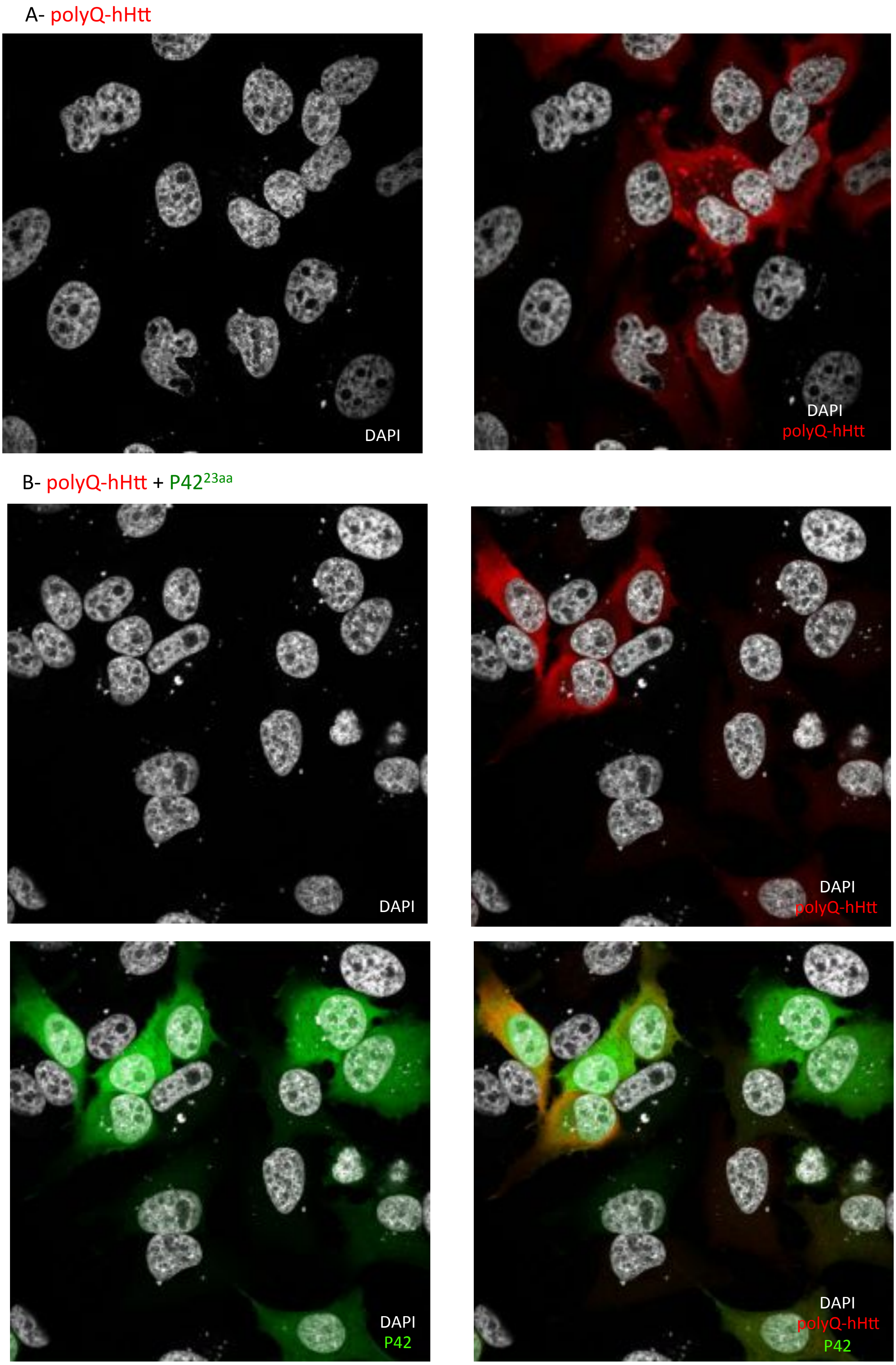

Figure S2: Low magnification images of HeLa cells transfected with polyQ-hHtt (in red). A- in absence of P42. B- in presence of P42 (in green). DAPI staining is shown in grey.
