# Supplementary material for: A Huntingtin Peptide Inhibits PolyQ-Huntingtin Associated Defects
Source: PLoS One. 2013 Jul 4;8(7):e68775. doi: 10.1371/journal.pone.0068775 (PMC3701666; doi:10.1371/journal.pone.0068775)
Supplement: Figure S3 — Alignment between dHtt (1-620aa) and hHtt P4 (166aa). (PDF) [file pone.0068775.s003.pdf]

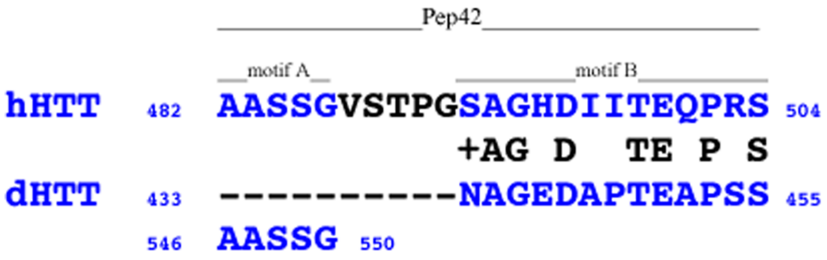

Figure S3: Alignment between dHtt (1-620aa) and hHtt P4 (166aa) (aa 382-548 region of hHtt). Using BLAStp NCBI, we identified a 13aa peptide showing 54% identities (motif B).
The AASSG motif (motif A) was identified in both dHtt and hHtt. In the human counterpart, A and B motifs of homologies are contained in a short 23aa peptide, referred as to P42.
Because A motif could be of importance, P42, containing both motifs, was designed and analysed for its protective properties.
