# Supplementary material for: A Huntingtin Peptide Inhibits PolyQ-Huntingtin Associated Defects
Source: PLoS One. 2013 Jul 4;8(7):e68775. doi: 10.1371/journal.pone.0068775 (PMC3701666; doi:10.1371/journal.pone.0068775)
Supplement: Figure S4 — Quantification of aggregates, using ImageJ software on confocal stacks. (PDF) [file pone.0068775.s004.pdf]

A- Quantification of aggregates in HeLa cells

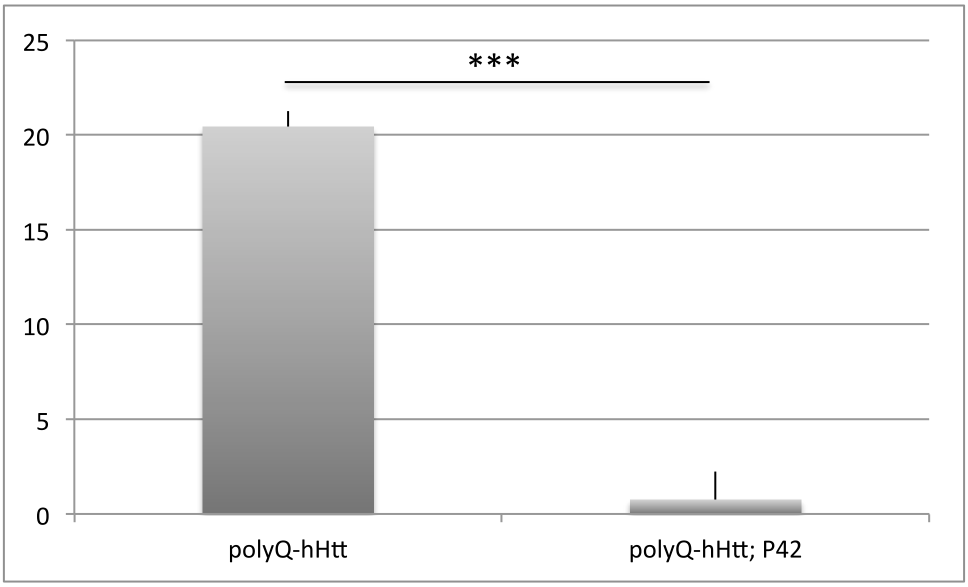

n=10-16 cells

B- Aggregate number on 10^4 μm^2 in salivary glands

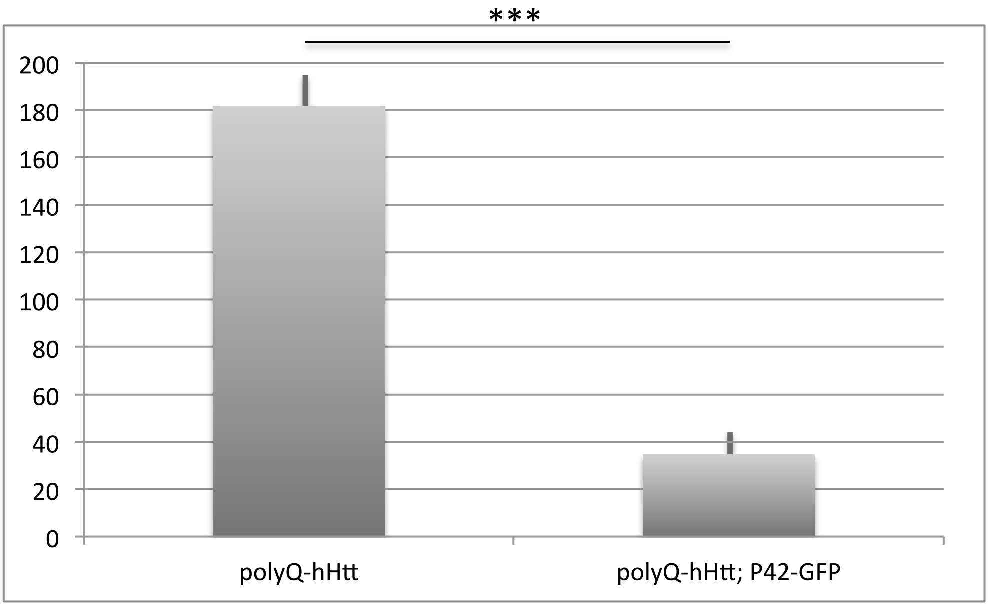

n=4-6 slices in 4 larvae

Figure S4: Quantification of aggregates, using ImageJ software on confocal stacks.
All images were threshold at the same intensity (120). We used the « analyze particles » function with a minimum size of 10 pixels to obtain a total number of particles (aggregates). Aggregates in absence (polyQ-hHtt) or in presence of P42 (polyQ-hHtt; P42) have been quantified in HeLa cells (A) and in larval salivary glands (B). Data were analysed by using the *Student's t-test*: ****p<0.001*.
