# Supplementary material for: A Huntingtin Peptide Inhibits PolyQ-Huntingtin Associated Defects
Source: PLoS One. 2013 Jul 4;8(7):e68775. doi: 10.1371/journal.pone.0068775 (PMC3701666; doi:10.1371/journal.pone.0068775)
Supplement: Figure S5 — PolyQ-hHtt expression in HeLa cells. (PDF) [file pone.0068775.s005.pdf]

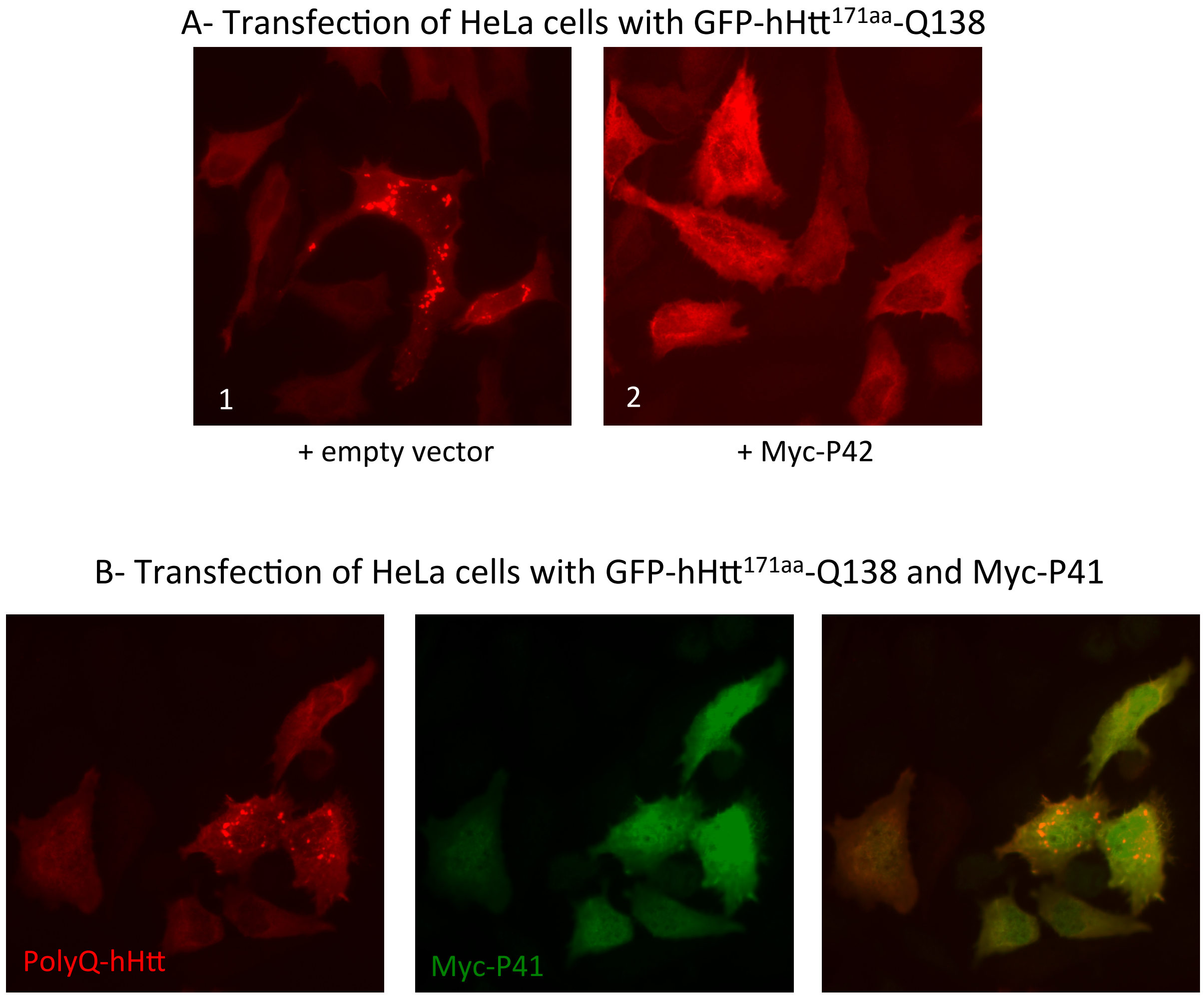

Figure S5: PolyQ-hHtt expression in HeLa cells (in red). (A1) in presence of BS empty vector. (A2) In presence of the P42 peptide Myc-tagged at its 5’ end: polyQ-hHtt do not form aggregates anymore. (B) In a same set of experiments, in presence of P41 peptide Myc-tagged at its 5’ end (in green), polyQ-hHtt (in red) still form aggregates.
