# Supplementary material for: A Huntingtin Peptide Inhibits PolyQ-Huntingtin Associated Defects
Source: PLoS One. 2013 Jul 4;8(7):e68775. doi: 10.1371/journal.pone.0068775 (PMC3701666; doi:10.1371/journal.pone.0068775)
Supplement: Figure S6 — In salivary glands, hHtt intensity and pattern does not change in presence of P42. (PDF) [file pone.0068775.s006.pdf]

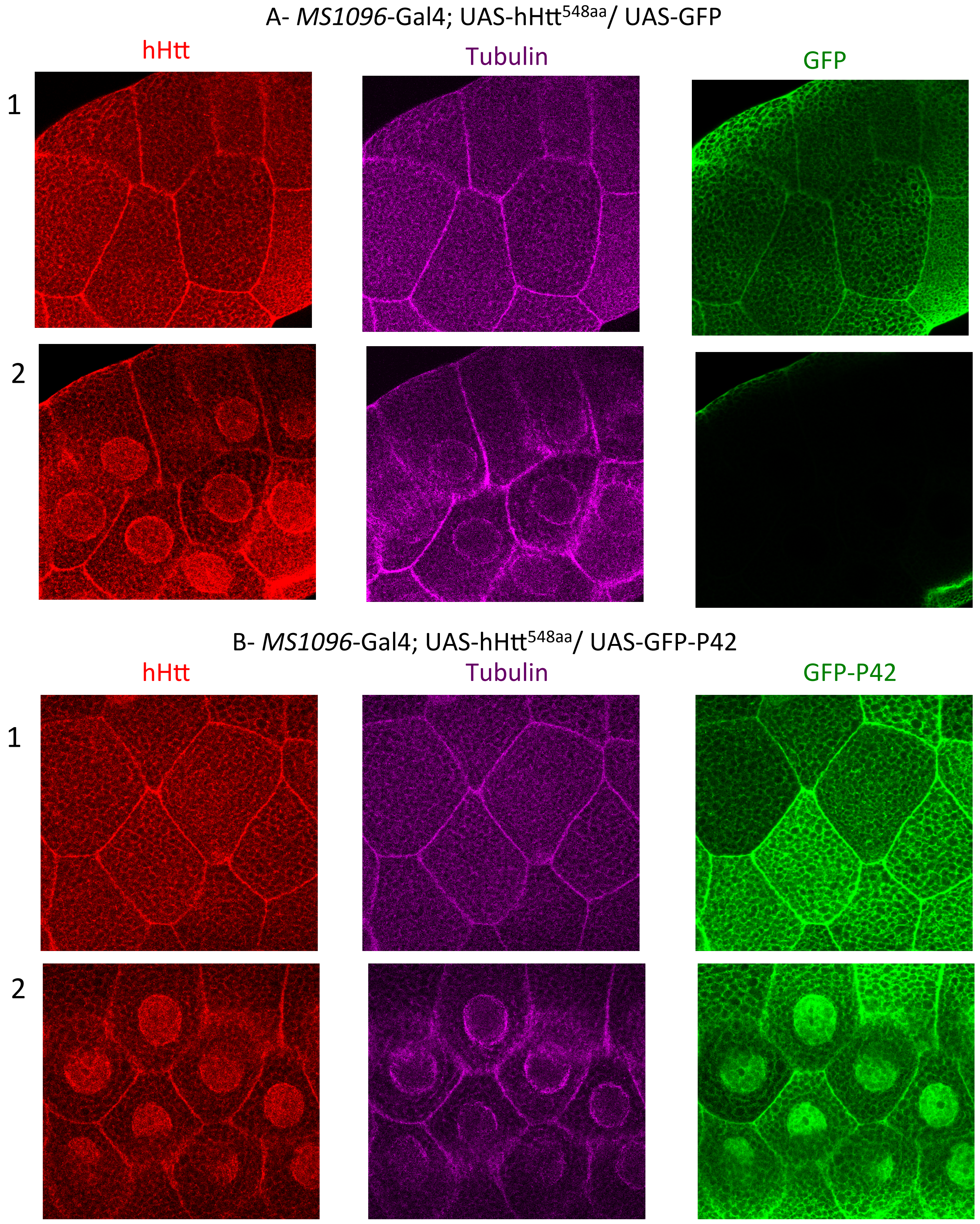

Figure S6: In salivary glands, hHtt intensity and pattern does not change in presence of P42. Human Htt staining pattern (in red) is similar to the Tubulin network (Magenta), at the cell periphery (A1). Neither pattern (hHtt and Tubulin) is modified in presence of P42 (B1). hHtt is also found in nuclei (A2). This localization is not modified in presence of P42 (B2), that is present in the same cellular compartments as hHtt. Same laser intensity has been used in A and B, confirming that hHtt intensity keeps also unchanged in presence of P42 .
