# Supplementary material for: A Huntingtin Peptide Inhibits PolyQ-Huntingtin Associated Defects
Source: PLoS One. 2013 Jul 4;8(7):e68775. doi: 10.1371/journal.pone.0068775 (PMC3701666; doi:10.1371/journal.pone.0068775)
Supplement: Figure S7 — Confocal images of third larval peripheral nerves expressing hHtt548aa. (PDF) [file pone.0068775.s007.pdf]

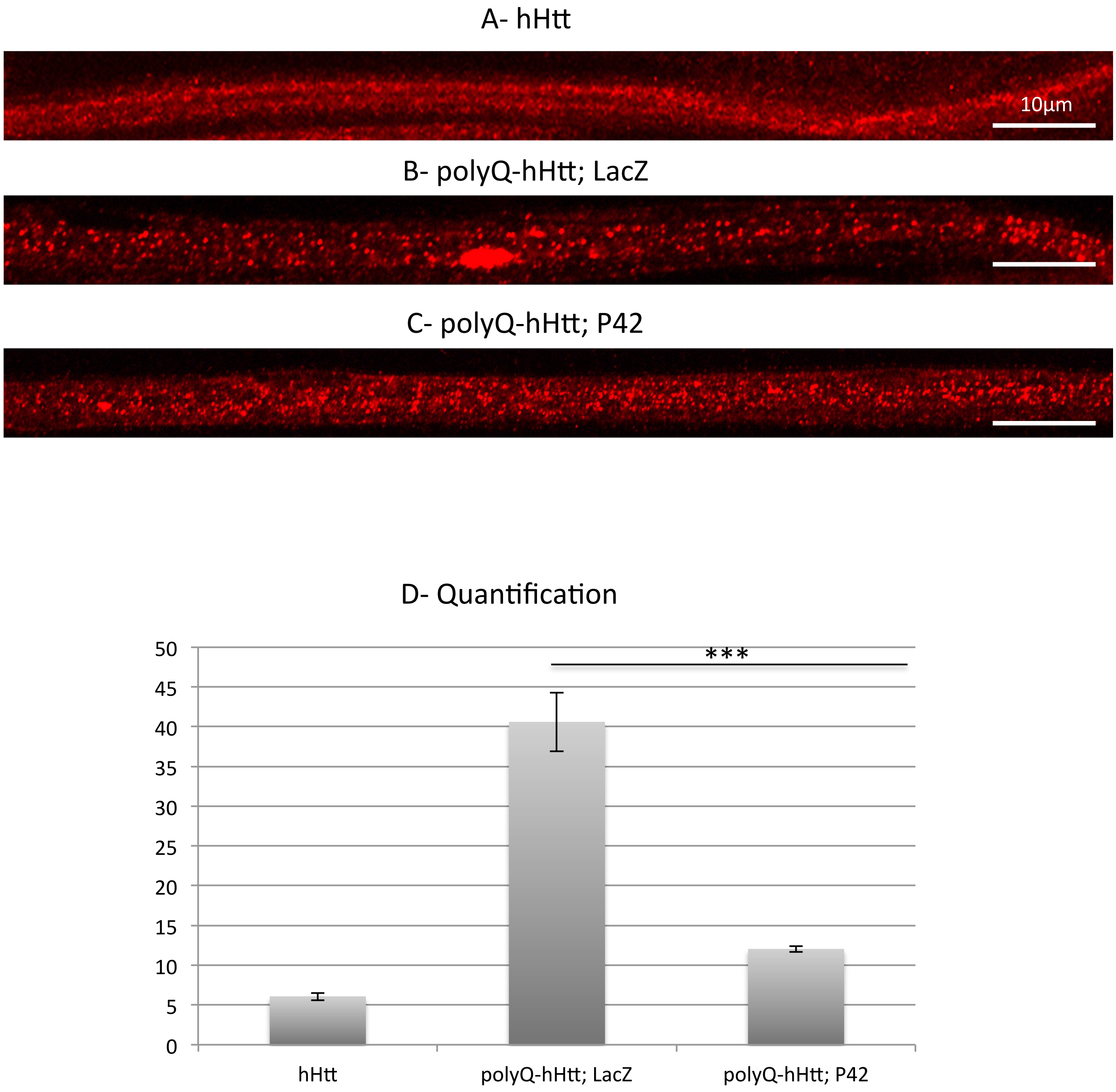

Figure S7: Confocal images of third larval peripheral nerves expressing $hHtt^{548aa}$, detected by anti-hHtt (Hu-4C8) (in red) expressing in A- OK6-Gal4; UAS-0Q-$hHtt^{548aa}$ (hHtt). B- OK6-Gal4; UAS-128Q-$hHtt^{548aa}$; UAS-LacZ. C- OK6-Gal4; UAS-128Q-$hHtt^{548aa}$; UAS-P42. D- Quantification of particle density for 100µm axon segments (*n=10*), using ImageJ particle quantification Plugin. Data were analysed by using the *Student's t-test*: ****p<0.001*.
