# Supplementary material for: A Huntingtin Peptide Inhibits PolyQ-Huntingtin Associated Defects
Source: PLoS One. 2013 Jul 4;8(7):e68775. doi: 10.1371/journal.pone.0068775 (PMC3701666; doi:10.1371/journal.pone.0068775)
Supplement: Figure S8 — Quantification of HHtt immunoreactivity at the NMJ. (PDF) [file pone.0068775.s008.pdf]

Ok6 Gal4/ UAS-NPY-GFP

1- polyQ-hHtt; LacZ

2- polyQ-hHtt; P42

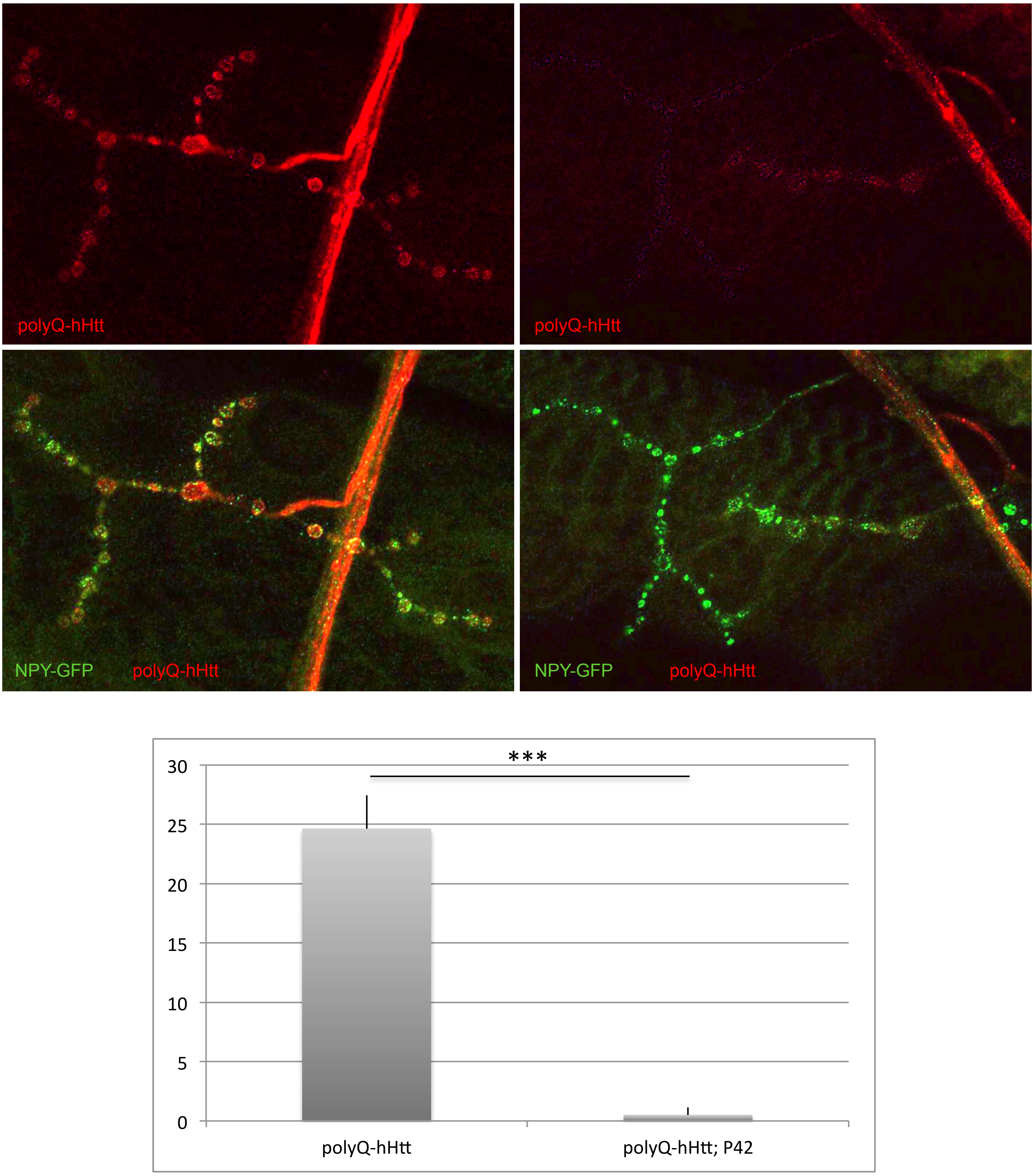

n=4-6 slices in 3 NMJ

Figure S8: Quantification of HHtt immunoreactivity (in red) at the NMJ, in absence (polyQ-hHtt) or in presence of P42 (polyQ-hHtt; P42). Data were analyzed by using the *Student's t-test*: ****p<0.001*.
