# Supplementary material for: A Huntingtin Peptide Inhibits PolyQ-Huntingtin Associated Defects
Source: PLoS One. 2013 Jul 4;8(7):e68775. doi: 10.1371/journal.pone.0068775 (PMC3701666; doi:10.1371/journal.pone.0068775)
Supplement: Figure S9 — Protein extracts from HeLa cells transfected by GFP-polyQ-hHtt. (PDF) [file pone.0068775.s009.pdf]

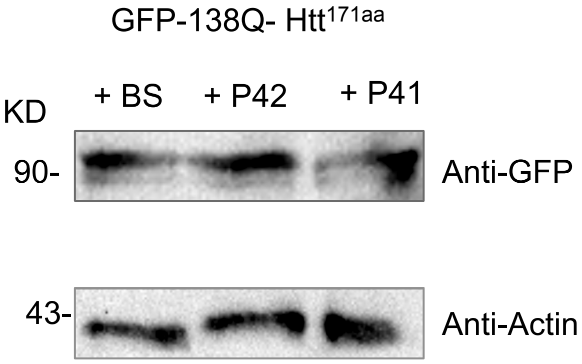

	+BS	+P42	+P41
**GFP**	100%	109%	113%
**Actin**	100%	118%	136%
**Ratio GFP/Actin**	1	0.92	0.83

Figure S9: Protein extracts from HeLa cells transfected by GFP-polyQ-hHtt in presence of empty vector (BS) or Cherry-P42 (P42) or Myc-P41 (P41) were analyzed on western blot. Note that when compared to Actin level of expression, the amount of expression of soluble polyQ-hHtt kept unchanged, ruling out that P42 is acting on Htt level of expression, which is confirmed in flies (Figure S6).

Quantifications have been performed on 2 independent experiments, which is summarized in the table: GFP and Actin were quantified relative to the control (+BS).
